# Detection of neuron specific enolase in the lip and ear pinna of goat (*Capra hircus*): an immunohistochemical study

**DOI:** 10.1186/s12917-025-05050-x

**Published:** 2025-10-03

**Authors:** Fatma EL-Zahraa Ahmed Mustafa

**Affiliations:** Department of Cell and Tissues, Faculty of Vet. Medicine, Assiut University, Assiut, 71526 Egypt

**Keywords:** Ruffini corpuscle, Goat corpuscle, Merkel cell, Fibroblast, Telocyte, Sinus hair, ENO2

## Abstract

Goat skin is considered a multifunctional organ with regulatory, sensory, and protective roles. Despite the goat’s economic importance and use as a model in biomedical research, it remains not given the suitable position in academic publications. This study aimed to investigate the expression of neuron-specific enolase (NSE) in the lips and ear pinnae of goats, revealing its considerable location among several sensory and structural components using immunohistochemical techniques. Also, we identified a new structure in different locations of the skin of the goat, referred to as the goat corpuscle. In the lip, NSE was strongly expressed in Ruffini corpuscles, goat corpuscles located in the skin and mucosa, and Merkel cells present in the stratum basale of the epidermis and hair follicles. NSE expression was also found in large nerves among skeletal muscle fibers, near sweat glands, sebaceous glands, and in the superficial layers of the dermis, where free nerve endings were directed. Vascular elements, including arteries, veins, and lymphatic vessels, exhibited NSE positivity in their endothelial and smooth muscle layers. Additionally, NSE was present in myoepithelial cells surrounding sweat and salivary gland secretory end pieces, fibroblasts, and telocytes in the connective tissue. In the ear, NSE was detected in Ruffini corpuscles, goat corpuscles, and a range of nerve fibers around cartilage and blood vessels. NSE expression was also observed in fibroblasts, chondroblasts, and telocytes in the cartilage and dermal tissues. This detailed mapping of NSE distribution provides new insights into its role in sensory and structural components, revealing its extensive involvement in the tissues.

**Graphical abstract:**

Immunohistochemical localization of NSE key observations in goat lip and ear pinna: A: Ruffini corpuscle (RC) near the hair follicle (H) and near the sebaceous gland (Sb). B: goat corpuscle in subepithelial position skin (white arrow), C: Merkel cell neurite complex (white arrowheads). D: Nerves (black arrows) nearly surrounded the sinus hair (H) from all sides. E: telocyte (yellow arrow) with long telopods (black arrowheads) near the sweat gland (Sw). F: Cartilage related cells as in fibroblast (blue arrowheads) in the perichondrium, chondroblast (black arrowhead), and peripherally located chondrocytes (red arrowheads). (A,B and C scale bar=100 μm; D scale bar=200 μm; E and F scale bar=50 μm)

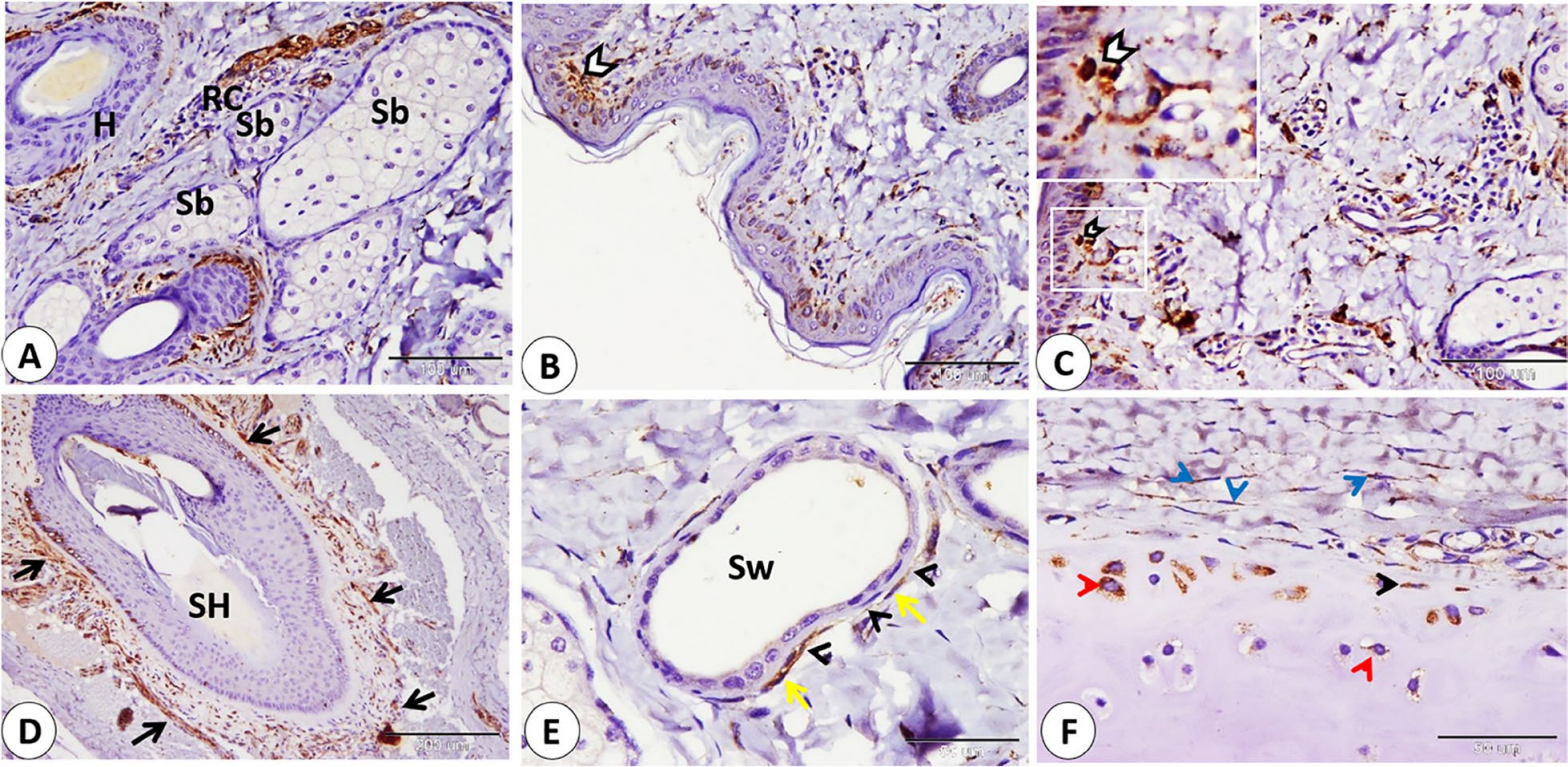

## Introduction

Skin is considered the body’s largest organ and primarily acts as a barrier between the body’s internal environment and the external environment. Skin serves as an effective barrier that plays several roles in maintaining body homeostasis and providing protection, including external sensory awareness, immunological defense, excretion, thermoregulation, and wound healing [1, 2].

The skin of goats is an important basic raw material in the leather industry [3]. Histologically, the epidermis and dermis make up the skin of a goat. Keratinocytes and non-keratinocytes form the epidermis. Keratinocytes consist of stratum basale, one layer of basally located cells; stratum spinosum, several layers of polyhedral cells; stratum granulosum, a single layer of fusiform cells; and stratum corneum, the outermost layer [3]. Non-keratinocytes are three cell types that each perform a specific function, including Merkel cells, which act as skin neuroendocrine cells, Langerhans cells, resembling skin immune cells, and melanocytes, which act as sources of skin pigmentation [4, 5].

90% of the skin covering the body was hairy skin. Skin of mammals with sensory nerve endings resembling touch organs with sensory components; different types of sensory nerve endings and mechanical components; hair, hair follicle, sebaceous gland, and connective tissues of dermis. Also, animals have sinus or tactile hair, which is located in different places in the animal’s body but is most commonly detected on the lips [6–8].

It has been established that neuron-specific enolase (NSE), also known as ENO2, is an isoenzyme of the glycolytic enzyme enolase. NSE is a marker that is extremely specific for neurons and neuroendocrine cells. Immunostaining techniques reveal the presence of NSE in several types of neurons, including granule cells, projection neurons, Purkinje cells, as well as autonomic and sensory neurons. It has similarly been observed in various types of healthy cells, such as telocytes, Merkel’s cells of the skin, adrenal medullary chromaffin cells, thyroid parafollicular cells, pituitary glandular and peptide-secreting cells, pinealocytes, cells of the islets of Langerhans, and neuroendocrine cells of the vagina and lung, as well as erythrocytes. According to the NSE findings in particular tissues under normal conditions, it was postulated that malignant proliferation in these tissues could result in elevated expression of NSE and increased levels of NSE in the bloodstream. This suggests that NSE could be useful in diagnosing, staging, and treating these types of cancer [9–13].

Goats are used as models in stem cell research and biomedical research and for studying diseases of humans and their management. Additionally, goats are used as educational models. They are small, reared in small areas, and easily managed [14–16]. Goats produce a variety of products, including high-nutrition milk and meat, fiber, and skin products, making them one of the most economically important animals. The goats are not given a suitable position in academic publications despite their economic importance [17]. So, in the present investigation, we detect the NSE immunohistochemically in the goat lip and ear pinna.

## Materials and methods

### Sample collection

In this study, three goats (*Capra hircus*) were obtained from a slaughterhouse. After the slaughter, which was conducted by Islamic practices, samples of the lip and ear were immediately collected and preserved in 10% formalin [18]. The research was approved by the Ethics Committee of the Faculty of Veterinary Medicine at Assiut University (No.06/2024/0249). All procedures were carried out in compliance with the applicable policies and guidelines.

### Immunohistochemistry

We initially dehydrate the samples with ethyl alcohol, followed by clearing them with methyl benzoate. The samples are then embedded in paraffin wax as the embedding medium. Using a microtome, we cut the samples into sections with a thickness of 3–5 micrometers [19].

The sections, each 3–5 micrometers thick, were first dewaxed and then rehydrated with alcohol. They were subsequently washed with PBS (pH 7.4). To inhibit endogenous peroxidase activity, we applied a 1% hydrogen peroxide block. The samples were then thoroughly rinsed under running water for 10 min and treated with 10 mM sodium citrate buffer (pH 6.0) and heated for 20 min in a water bath to 95–98 °C to enhance antigen retrieval. At room temperature, the sections were allowed to cool, after which they were washed with PBS. To prevent non-specific background staining, an Ultra V block was applied. The primary antibody used was NSE (1:100; Catalog # PA5-27452). Mayer’s hematoxylin was employed as a counterstain. Images were captured using an OLYMPUS DP72 camera mounted on an OLYMPUS BX51 microscope [20, 21].

## Results

The current research describes the expression of NSE in the lip and ear pinna of goats. NSE was demonstrated in mechanoreceptors, including encapsulated receptors (Ruffini corpuscle), non-encapsulated receptors (Merkel cell), and free nerve endings. Also, NSE expression is described at different structural components as nerves, hair, hair follicles, sebaceous glands, sweat glands, salivary glands, muscles, vessels, cartilage, and interstitial cells.

### NSE expression in the lip

**Fig. 1:**
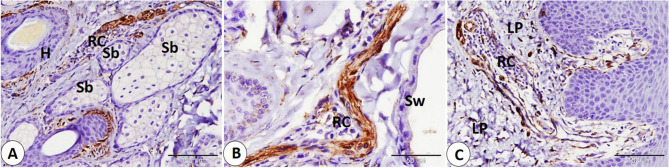
Immunohistochemical localization of NSE in the goat lip showed Ruffini corpuscle (RC) in the skin and the mucosa of the lip. **A** Ruffini corpuscle (RC) near the hair follicle (H) and near the sebaceous gland (Sb). **B** Ruffini corpuscle (RC) near the sweat gland (Sw). **C** Ruffini corpuscle (RC) in the lamina propria (LP). (**A** and **C** scale bar = 100 μm; **B** scale bar = 50 μm)

**Fig. 2:**
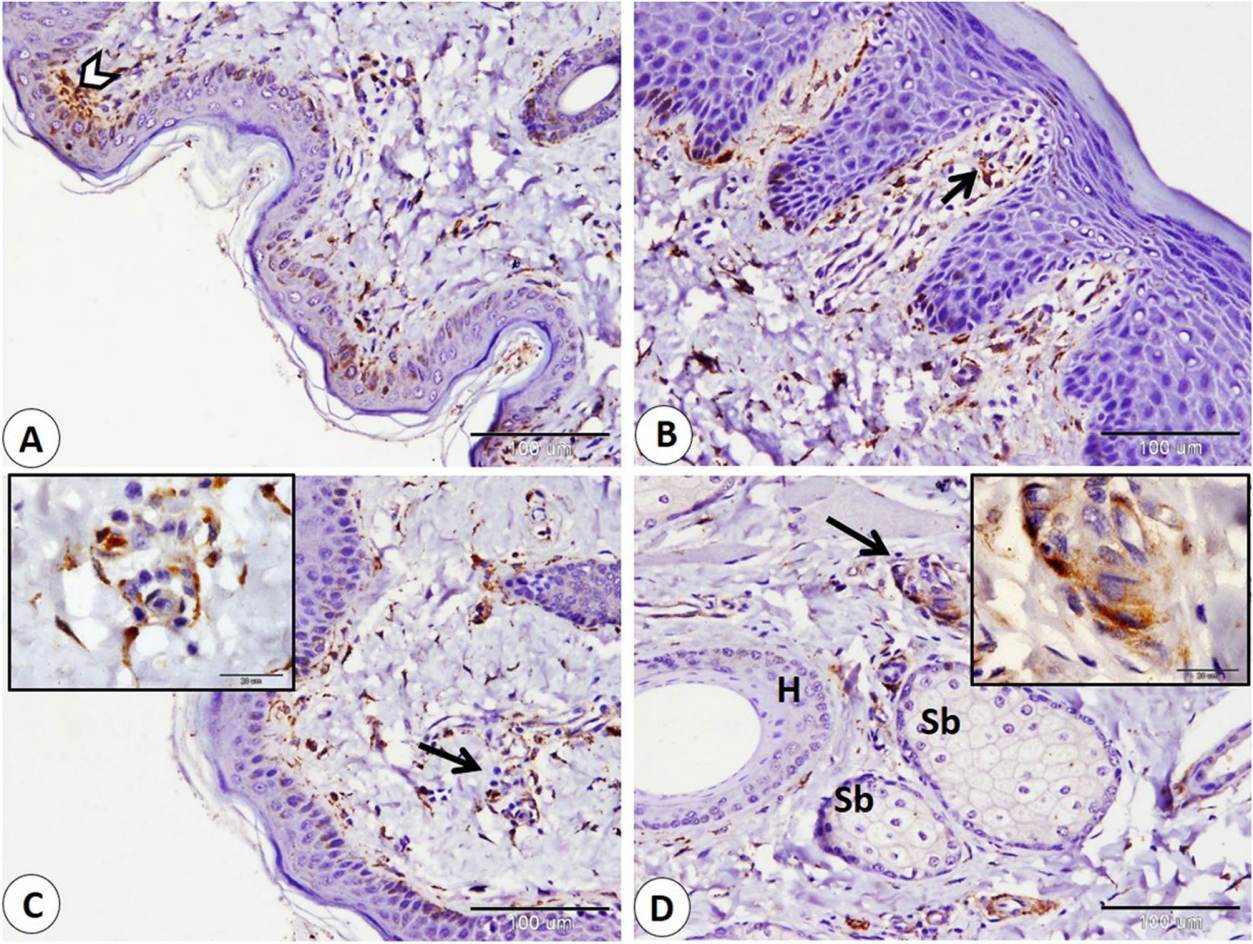
NSE immunohistochemical localization in goat lip showed goat corpuscle. **A**, **B** goat corpuscle in subepithelial position skin (white arrowhead), and mucosa (black arrow). **C**: goat corpuscle in reticular layer of dermis (black arrow), and the bordered area in **C** is a magnification of the goat corpuscle. **D** goat corpuscle (black arrow) near hair follicles (H), and sebaceous gland (Sb), and the bordered area in **D** is a magnification of the goat corpuscle. (**A**,** B**,**C** and **D** scale bar = 100 μm; bordered areas scale bar = 20 μm)

**Fig. 3:**
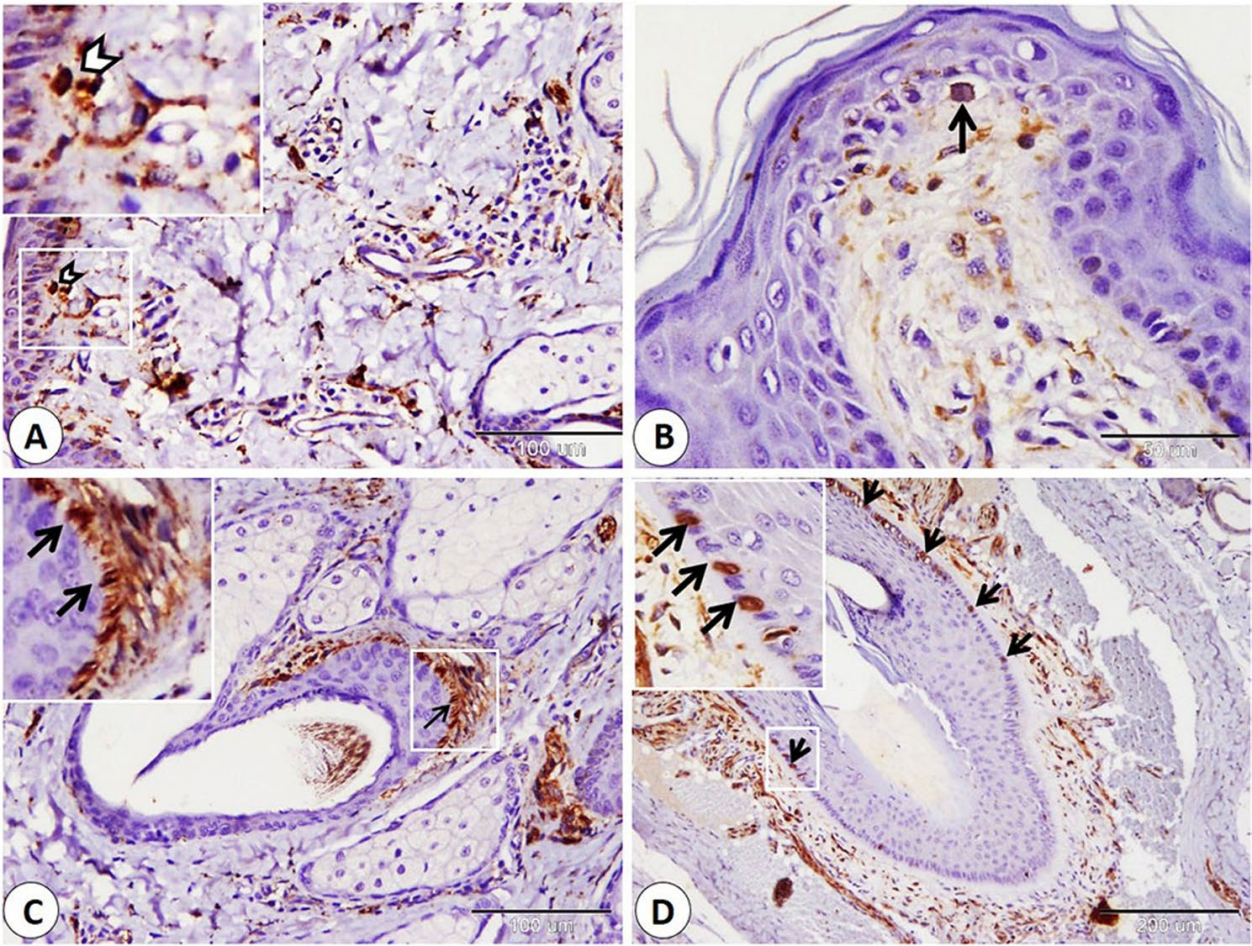
Immunohistochemical localization of NSE in the Merkel cell. **A**,** B** Merkel cell neurite complex (white arrowheads), and Merkel cell (black arrow) in the skin of the lip. **C** Merkel cell in hair follicle (black arrows). **D** numerous Merkel cells in the sinus hair follicle (black arrows). (**A** and **C** scale bar = 100 μm; **B** scale bar = 50 μm; **D** scale bar = 200 μm)

The Ruffini corpuscle is considered an encapsulated sensory corpuscle that exhibits NSE-positive expression and is observed on both surfaces of the lip, skin, and mucosa. Ruffini corpuscles are distributed in the dermis near the hair follicle, sebaceous gland, and sweat gland. Additionally, we observe it in the lamina propria (Fig. 1A-B-C). Moreover, we primarily observe small, rounded to oval, encapsulated corpuscles that were first identified and named as goat corpuscles; they give positive NSE expression and are located in subepithelial positions on both the skin and mucosa surfaces, as well as in the reticular layer of the dermis, near hair follicles, and sebaceous glands (Fig. 2A-B-C-D). In addition, the Merkel cell or Merkel cell neurite complex showed positive NSE expression and is detected in the skin of the lip, hair follicle, and sinus hair follicle. Merkel cells appear numerous in the follicle of the sinus hair, especially in the upper half (Fig. 3A-B-C-D).

**Fig. 4:**
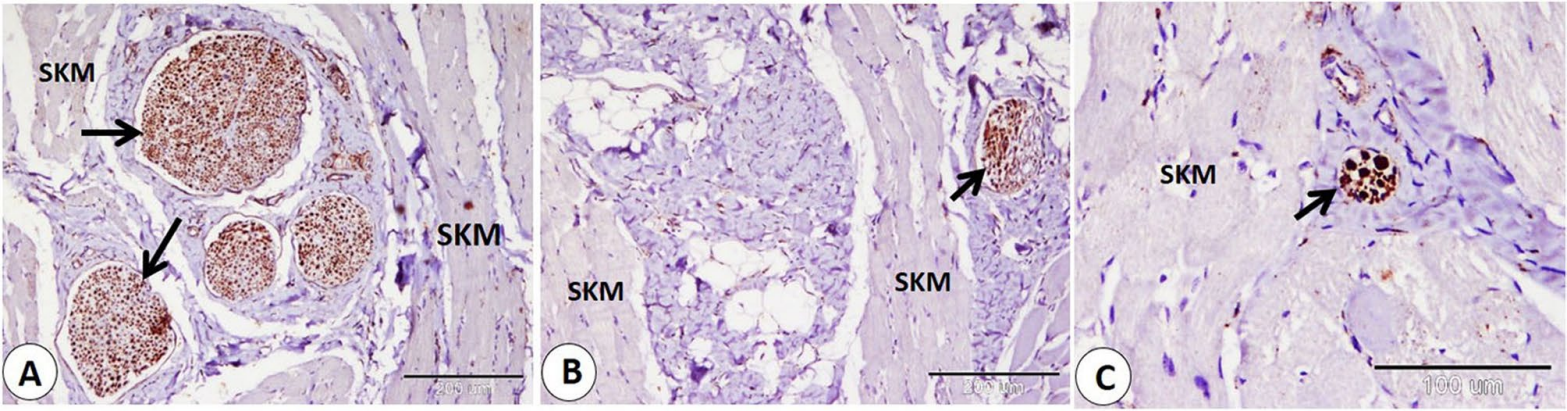
Immunohistochemical localization of NSE in nerves of goat lip. **A** large nerves (black arrows) between skeletal muscles (SKM). **B**, **C** smaller branches of nerves (black arrows) between muscles (SKM). (**A** and **B** scale bar = 200 μm; **C** scale bar = 100 μm)

**Fig. 5:**
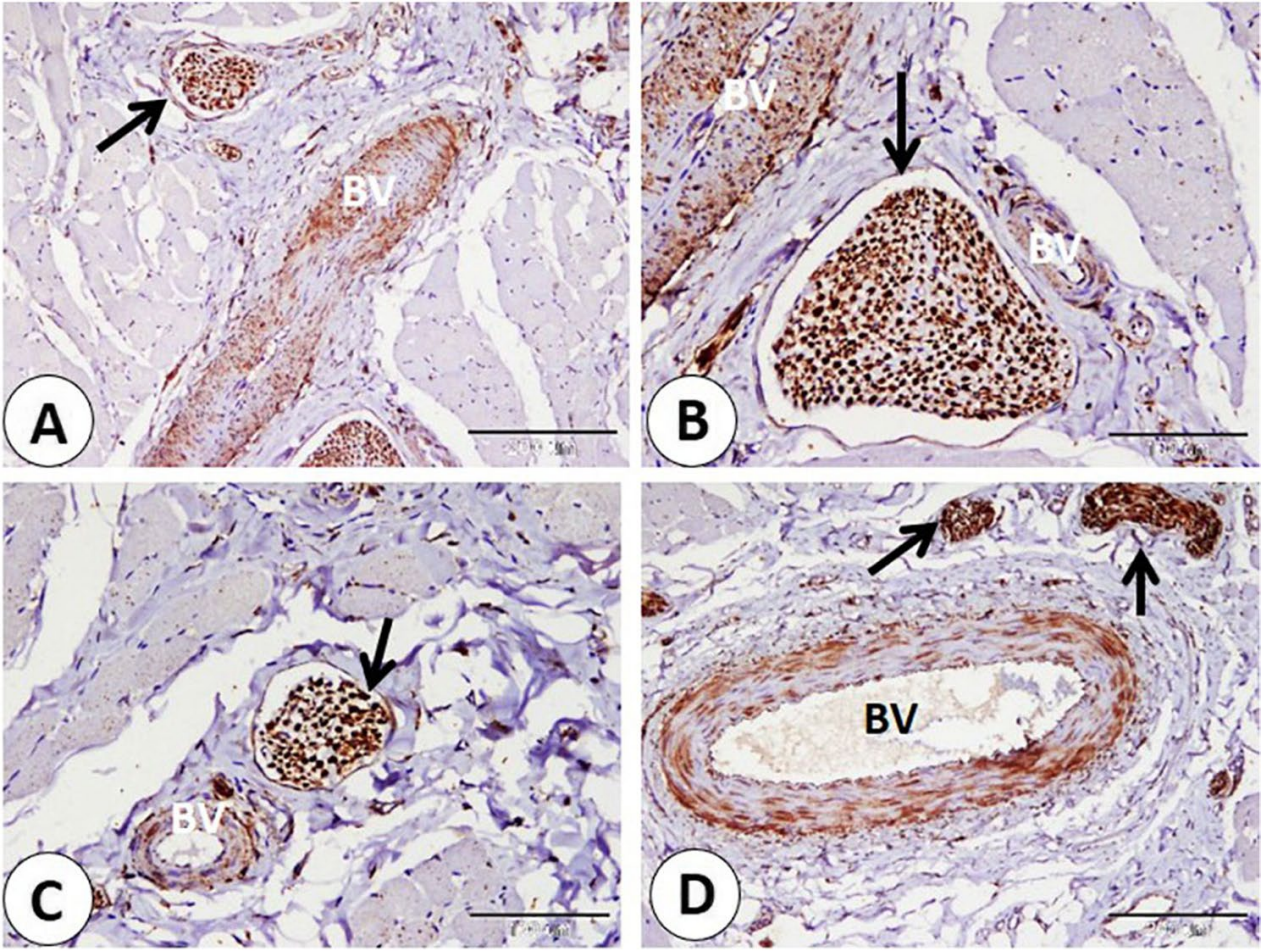
**A-B-C-D** Showed immunohistochemical localization of NSE in nerves (black arrows) of different sizes in goat lip near blood vessels of different sizes (BV). (**A** and **D** scale bar = 200 μm; **B** and **C** scale bar = 100 μm)

**Fig. 6:**
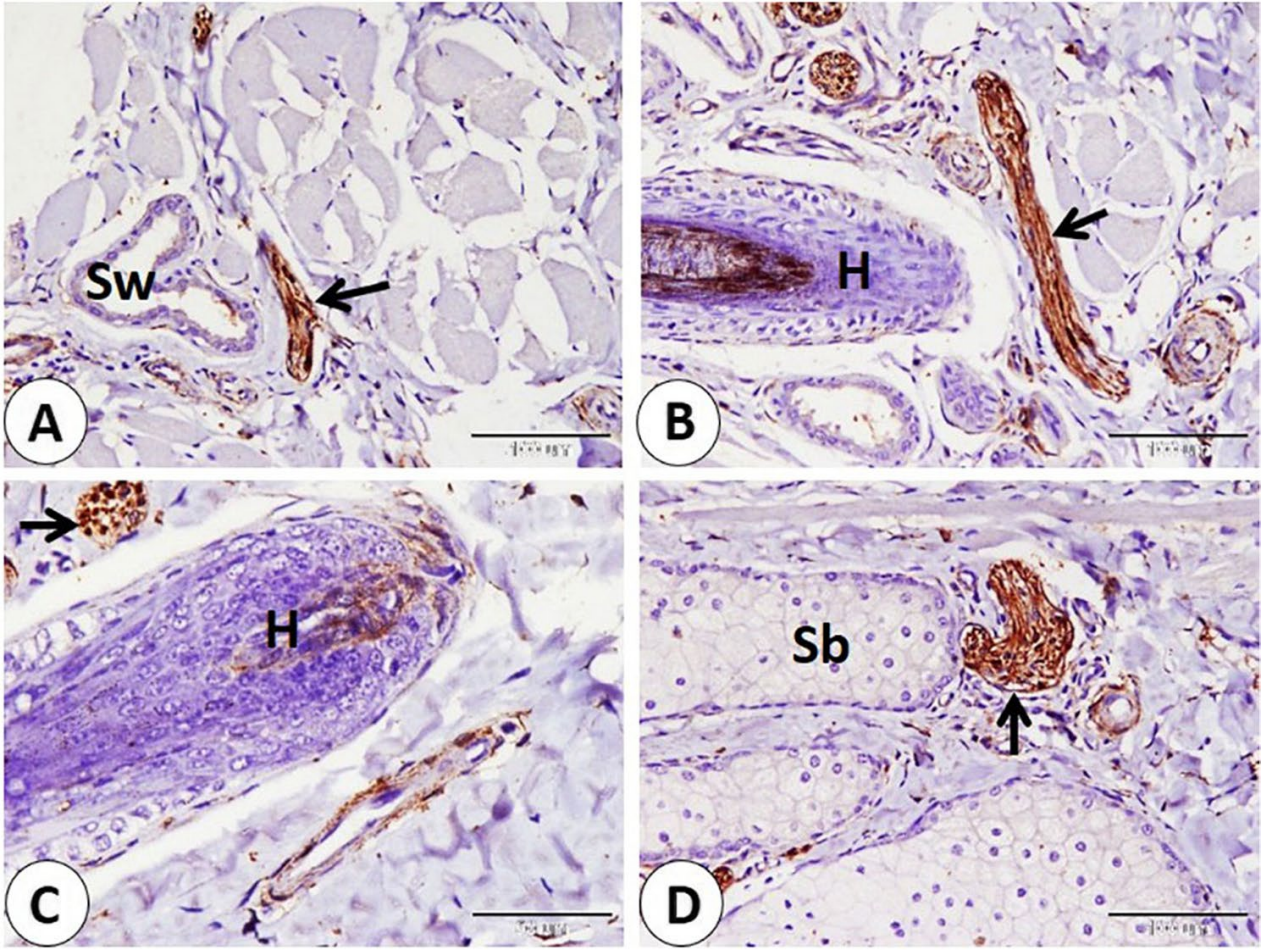
Immunohistochemical localization of NSE in nerves which directed superficially at goat lip: **A** nerves (black arrow) near sweat gland (Sw). **B-C** nerves (black arrows) near the hair (H). **D** nerves (black arrow) near sebaceous gland (Sb). (**A**,** C** and **D** scale bar = 100 μm; **B** scale bar = 50 μm)

**Fig. 7:**
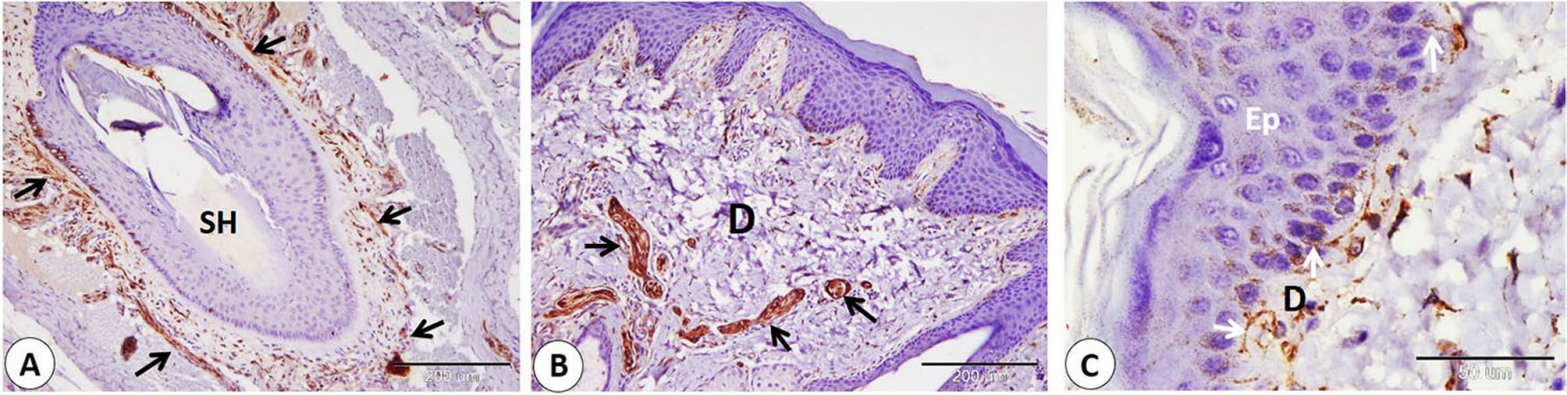
NSE immunohistochemical localization in goat nerves which directed superficially at goat lip. A: Nerves (black arrows) nearly surrounded the sinus hair (H) from all sides. **B** Large amount of nerves (black arrows) detected on the superficial layer of the reticular dermis (D). **C** Nerves directed toward papillary dermis (D) forming the free nerve ending (white arrows) in the epidermis (Ep). (**A** and **B** scale bar = 200 μm; **C** scale bar = 50 μm)

**Fig. 8:**
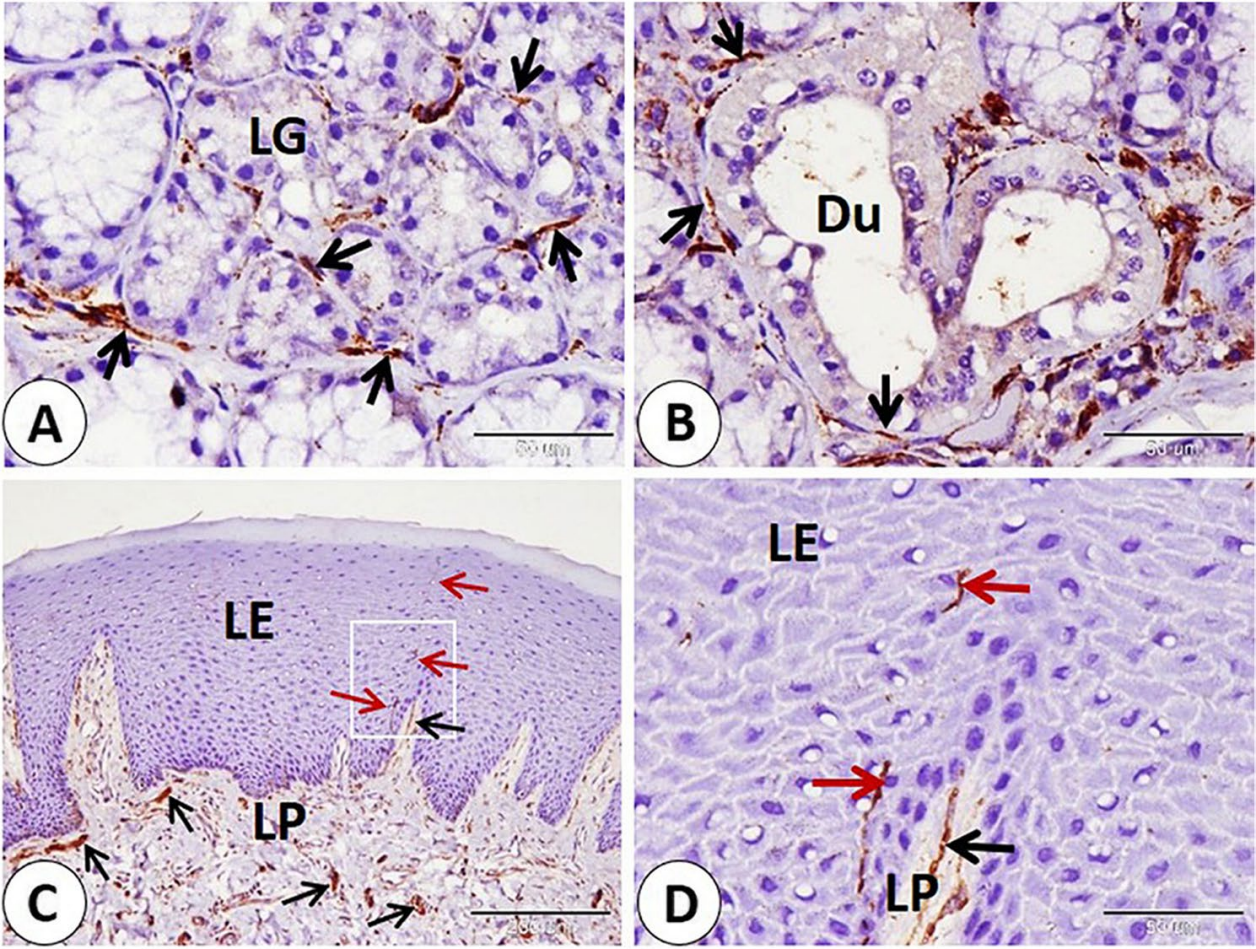
Immunohistochemical localization of NSE in nerves located on mucosal surface of the lip of the goat. **A**,** B** nerves (black arrows) surrounded secretory end pieces of the labial gland (LG) and ducts (Du). **C**,** D **(**D: **magnification of bordered area in **C**): large amount of nerves (black arrows) on the lamina propria (LP) directed toward the lamina epithelilais (LE). nerves forming free nerve ending which reach until superficial layers of the epithelium (red arrows). (**A**,** B** and **D** scale bar = 50 μm; **C** scale bar = 200 μm)

We observed a series of nerves of different sizes that showed NSE-positive expression. The largest nerves were observed deep between skeletal muscle fibers. Nerves were given smaller branches between muscle fibers (Fig. 4A-B-C-D). Also, large and small nerves detect near-important structural components as blood vessels of different sizes (Fig. 5A-B-C-D). Afterward the nerves directed superficially near the sweat gland, near the hair bulb, and the sebaceous gland (Fig. 6A-B-C-D). Nerves nearly covered the sinus hair from all sides (Fig. 7A). A large amount of nerves was detected on the superficial layer of the reticular dermis, which was directed toward the papillary dermis, forming the free nerve ending in the epidermis, which was considered non-encapsulated receptors (Fig. 7B-C). On the opposite surface, nerves were observed surrounding secretory end pieces of the labial gland and their ducts, and then a large amount of nerves were observed on the lamina propria directed toward the lamina epithelialis, forming the free nerve ending, which reached until the superficial layers of the epithelium (Fig. 8A-B-C-D).

**Fig. 9:**
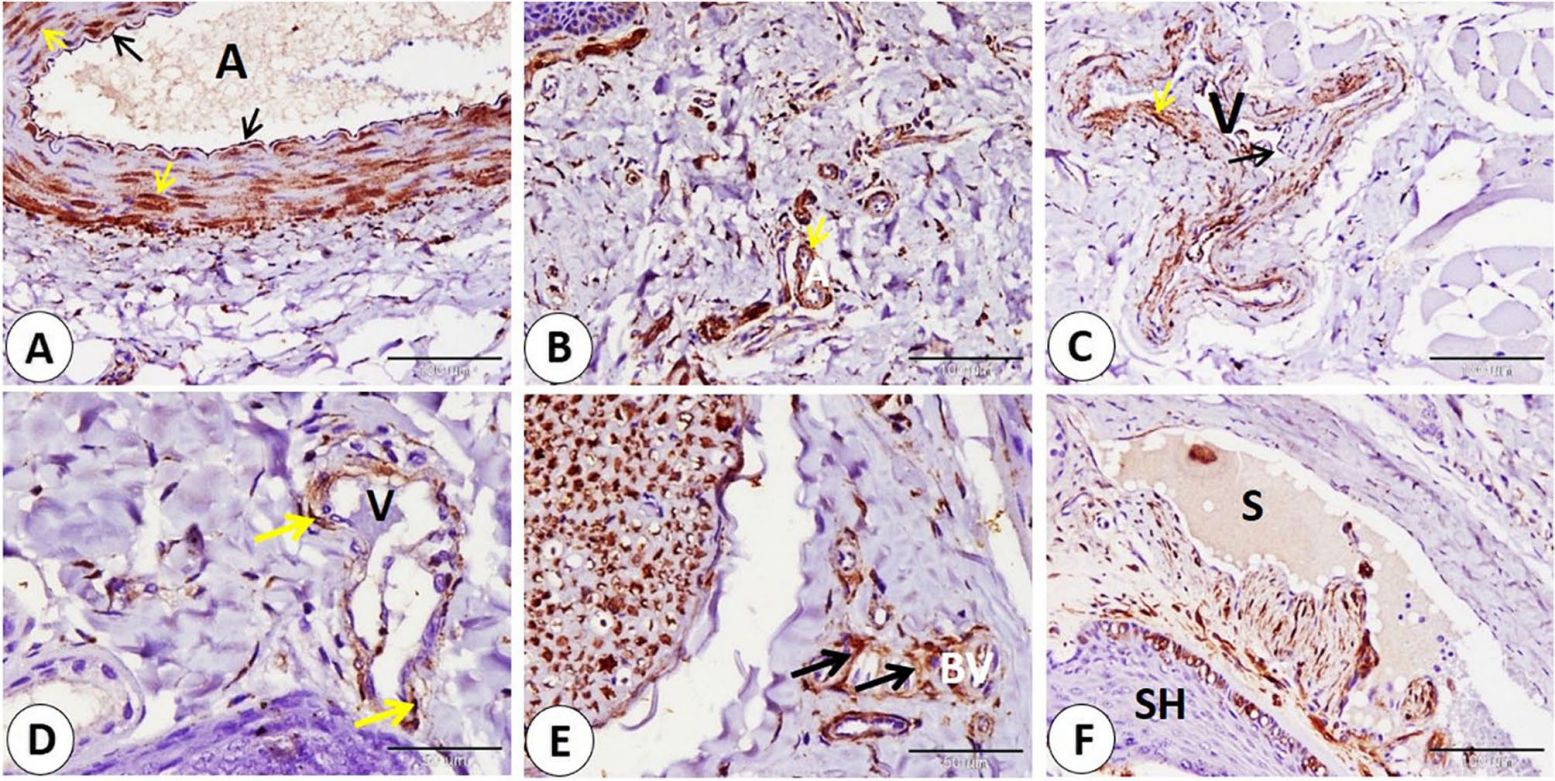
Immunohistochemical localization of NSE in goat lip vascular elements. **A**,** B** arteries of different sizes (A) exhibited NSE positive expression in different parts including endothelium (black arrows), muscular layer (yellow arrows). **C**,** D** veins of different sizes (V) exhibited NSE positive expression in different parts including endothelium (black arrows) muscular layer (yellow arrows). **E** NSE expressed in middle layer of epitheloid cells (black arrows) in special type of blood vessels (BV). **F** NSE expressed in in plasma (P) present in sinuses of sinus hair (SH). (**A**,** B**,**C** and **F** scale bar = 100 μm; **D** and **E** scale bar = 50 μm)

Vascular elements—arteries and veins—of various sizes exhibited NSE-positive expression in different parts, including the endothelium muscular layer. In addition, NSE is expressed in the middle layer of epithelioid cells in special types of blood vessels present near large nerves and in plasma present in sinuses of sinus hair (Fig. 9A-B-C-D-E-F).

**Fig. 10:**
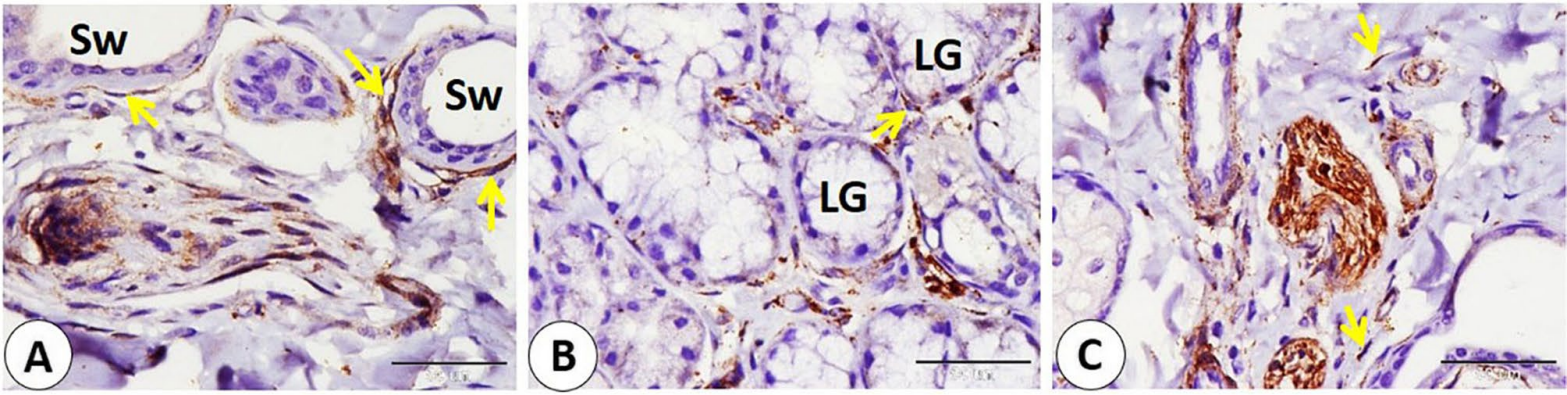
Immunohistochemical localization of NSE in different important cellular component in the goat lip. **A** NSE expression in myoepithelial cells (yellow arrows) surrounded sweat gland secretory end pieces (Sw). **B** NSE expression in myoepithelial cells (yellow arrow) surrounded salivary gland secretory end pieces (LG). **C**: NSE expression in fibroblast (yellow arrows) in the connective tissue. (**A**,** B** and **C** scale bar = 50 μm)

**Fig. 11:**
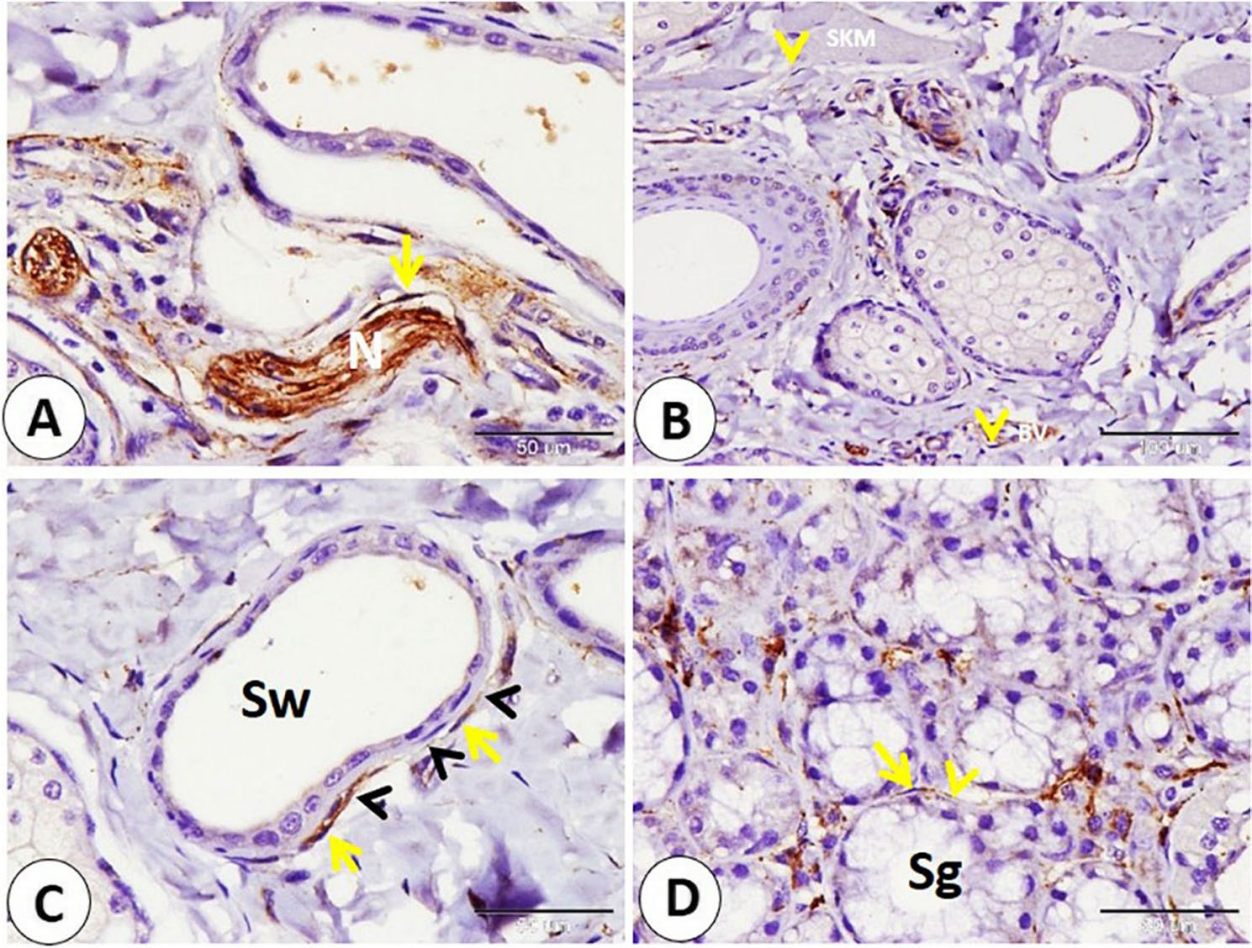
Immunohistochemical localization of NSE in telocyte in the goat lip. **A** telocyte (yellow arrow) near the nerve fibers (N). **B** telocyte (yellow arrowheads) near skeletal muscle fibers (SKM) and blood vessels (BV). **C** telocyte (yellow arrow) with long telopods (black arrowheads) near the sweat gland (Sw). **D** telocyte (yellow arrow) with long telopods (yellow arrowheads) near the salivery gland (Sg). (**A**,** C** and **D** scale bar = 50 μm; **B** scale bar = 100 μm)

Different important cellular components in the lip showed NSE expression, including myoepithelial cells surrounding the sweat gland secretory end pieces, myoepithelial cells surrounding salivary gland secretory end pieces, and fibroblasts in the connective tissue (Fig. 10A-B-C). In addition, telocytes with their characteristic small cell body and long cytoplasmic processes were detected near the nerve fibers, skeletal muscle fibers, blood vessels, and gland secretory end pieces (sweat and salivary) and showed NSE-positive reactivity (Fig. 11A-B-C-D).

### NSE expression in the ear

**Fig. 12:**
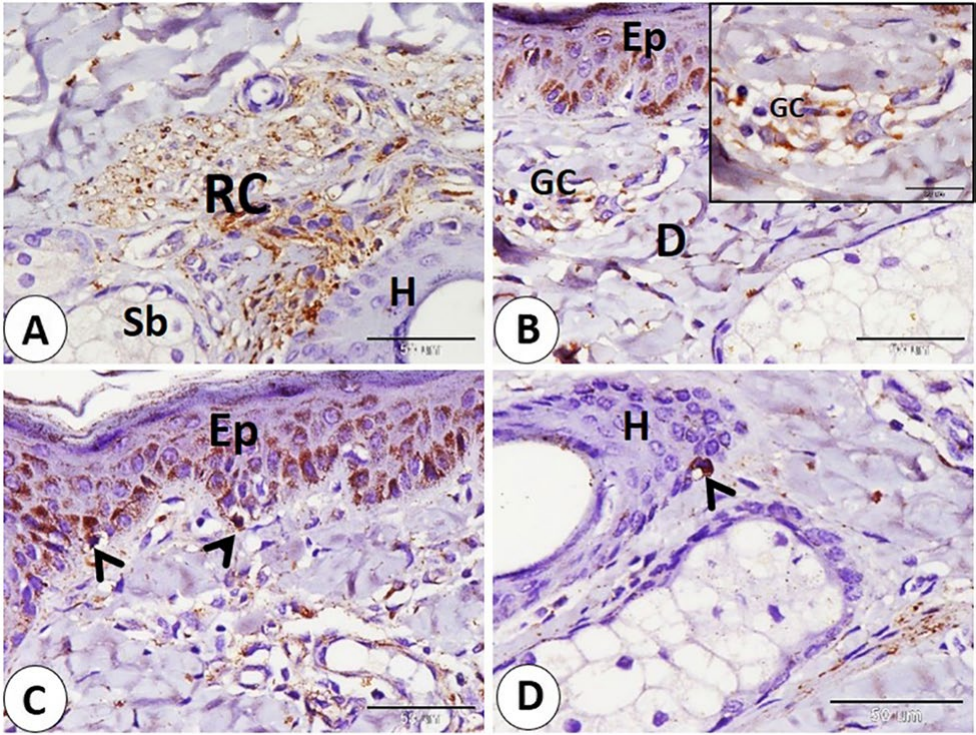
Immunohistochemical localization of NSE in goat ear skin showed Ruffini corpuscle, goat corpuscle, and Merkel cells. **A** Ruffini corpuscle (RC) near the hair follicle (H) and near the sebaceous gland (Sb). **B** goat corpuscles (GC) in dermis (D) near the epidermis (Ep), and the bordered area in B is a magnification of the goat corpuscle. **C**,** D**: Merkel cells (black arrows) in the epithelium (Ep), and on the hair follicle (H). (**A**,** B**,**C** and **D** scale bar = 50 μm; bordered area scale bar = 20 μm)

**Fig. 13:**
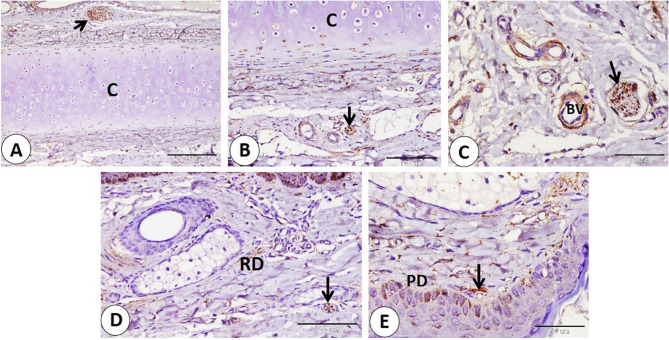
**A-B-C-D-E** Showed immunohistochemical localization of NSE in nerves (black arrows) of different sizes at different depth in goat ear near the cartilage (**C**), near blood vessels (BV) of different sizes, in reticular dermis (RD) in papillary dermis (PD). (**A** scale bar = 200 μm; **B** and **D** scale bar = 100 μm; **C** and **E** scale bar = 50 μm)

The Ruffini corpuscle was visible in the ear skin near the hair follicle and sebaceous gland (Fig. 12A). Additionally, subepithelial position in the dermis revealed the presence of small corpuscles, known as goat corpuscles (Fig. 12B). Several Merkel cells were detected in the epithelium of the skin between stratum basale cells, but fewer Merkel cells were found on the hair follicle (Fig. 12C-D). The largest nerves were observed deep near the cartilage and large blood vessels. Also, small nerves are distributed in different positions near cartilage and blood vessels in the dermis. In addition, nerve fibers are distributed in the reticular dermis in a subepithelial position (Fig. 13A-B-C-D-E).

**Fig. 14:**
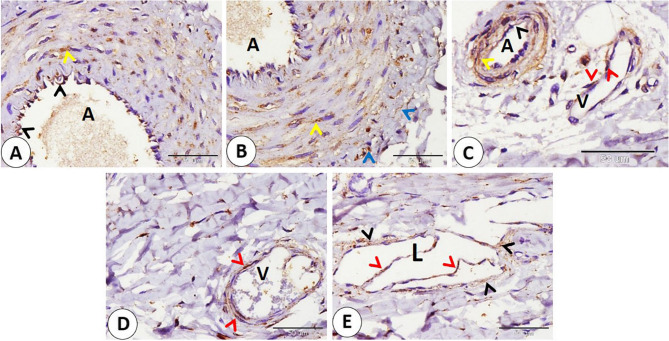
Immunohistochemical localization of NSE in goat ear Arteries, veins, lymph vessels. **A-B-C**: NSE expression in arteries (A) endothelium (black arrowheads), smooth muscle fibers (yellow arrowheads), and adventitia (blue arrowheads). **C**,** D** smooth muscles (red arrowheads) of veins (V) of different sizes expressed NSE. **E**: on lymphatic vessels (L) NSE expressed on valves (red arrowheads) and surrounding connective tissue (black arrowheads). (**A**,** B**,**C**,** D** and **E** scale bar = 50 μm)

The walls of arteries with varying sizes exhibited NSE expression in the endothelium, smooth muscle fibers in the middle layer, and adventitia. Also, veins of different sizes expressed NSE on the smooth muscles of their walls. Valves of lymphatic vessels and surrounding connective tissue of the lymph vessels expressed an NSE-positive reaction (Fig. 14A-B-C-D-E).

**Fig. 15:**
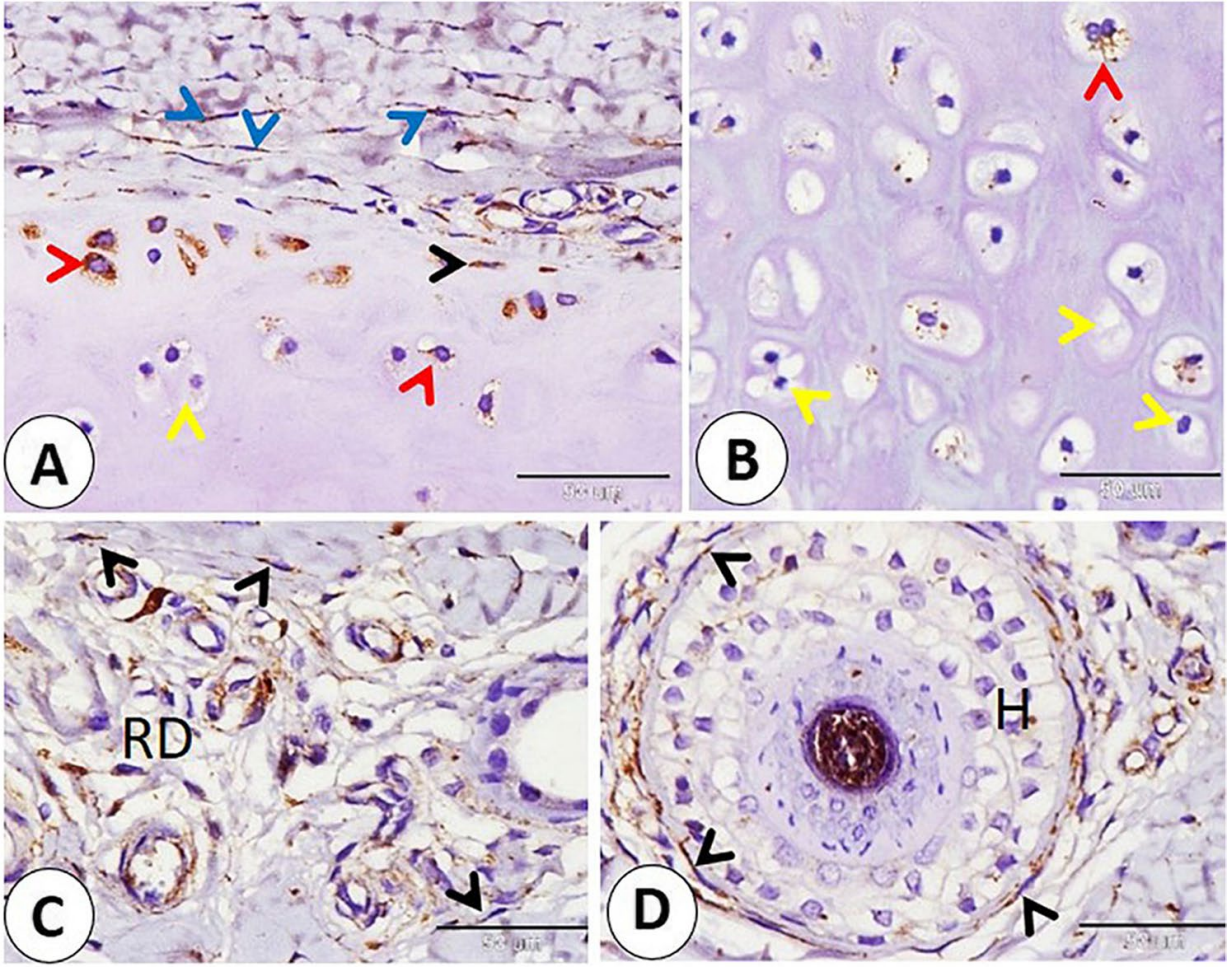
**A-B** showed immunohistochemical localization of NSE in Cartilage related cells as in fibroblast (blue arrowheads) in the perichondrium, chondroblast (black arrowhead), and peripherally located chondrocytes (red arrowheads). Negative NSE expression in deeply located chondrocytes (yellow arrowheads). **C**,** D** NSE positive reactivity in the fibroblast (black arrowheads) in the reticular dermis (RD) and in the hair follicle (H). (**A**,** B**,**C**,** D** and **E** scale bar = 50 μm)

**Fig. 16:**
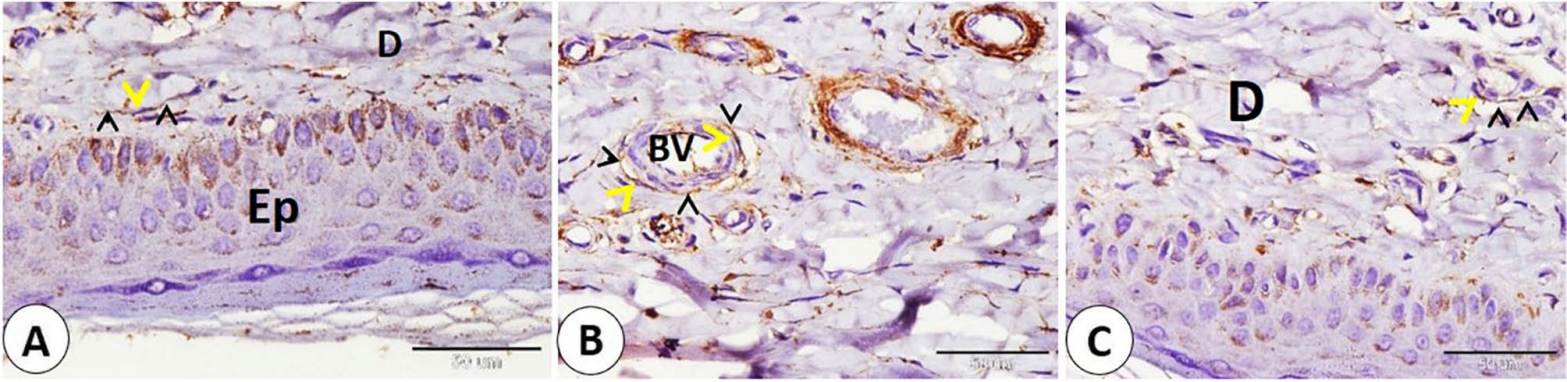
Immunohistochemical localization of NSE in telocyte in the goat ear. A: telocyte cell body (yellow arrowhead) and telopods (black arrowheads) in the dermis (D) near the epithelium (Ep). **B** telocyte cell body (yellow arrowheads) and telopods (black arrowheads) near the blood vessels (BV). **C** telocyte cell body (yellow arrowheads) and telopods (black arrowheads) near the nerve in the dermis (D). (**A**,** B** and **C** scale bar = 50 μm) Graphical abstract fig. Immunohistochemical localization of NSE key observations in goat lip and ear pinna: **A**: Ruffini corpuscle (RC) near the hair follicle (H) and near the sebaceous gland (Sb). **B**: goat corpuscle in subepithelial position skin (white arrow), C: Merkel cell neurite complex (white arrowheads). **D** Nerves (black arrows) nearly surrounded the sinus hair (H) from all sides. **E** telocyte (yellow arrow) with long telopods (black arrowheads) near the sweat gland (Sw). **F** Cartilage related cells as in fibroblast (blue arrowheads) in the perichondrium, chondroblast (black arrowhead), and peripherally located chondrocytes (red arrowheads). (**A**,** B** and **C** scale bar = 100 μm; **D** scale bar = 200 μm; **E** and **F** scale bar = 50 μm)

Cartilage-related cells such as fibroblasts in the perichondrium, chondroblasts, and peripherally located chondrocytes exhibited positive reactivity to NSE. On the other hand, NSE reactivity decreases in chondroblasts when going deep into the cartilage until it disappears (Fig. 15A-B). Fibroblasts in the reticular dermis and in the hair follicle showed NSE-positive reactivity (Fig. 15C-D). Also, telocytes near the blood vessels, nerve fibers, and subepithelial telocyte expressed an NSE-positive reaction (Fig. 16A-B-C).

## Discussion

This study represents, as far as we know, the first description of the expression of neuron-specific enolase in goats’ lip and ear pinna. Including different structural components of these parts: encapsulated receptors and non-encapsulated receptors, free nerve endings, hair, hair follicles, sebaceous glands, sweat glands, salivary glands, muscles, vessels, cartilage, and interstitial cells. Our data provides a mapping of NSE expression in key sensory structures such as Ruffini corpuscles, goat corpuscles, and Merkel cells. In addition, the expression is detected in free nerve endings and peripheral nerves. Moreover, NSE was detected in non-neuronal structures such as vascular components, fibroblasts, telocytes, chondroblasts, and myoepithelial cells.

Enolase, discovered by Lohmann and Meyerhof in 1934, is a key enzyme in glycolysis that converts 3-phosphoglycerate to pyruvate, generating ATP and NADH, crucial for cellular energy. The neuron-specific variant of this enzyme, known as neuron-specific enolase (NSE), was identified by Moore and McGregor in 1965 due to its distinct enolase activity. Enolases are vital for energy metabolism in a diverse range of organisms, from archaebacteria to mammals. Their expression levels can fluctuate depending on cellular energy needs, developmental stages, and various metabolic disorders [22–26].

The skin, which covers about 12% of the body, is the largest organ. It serves multiple crucial roles, such as protecting, maintaining temperature, and having economic significance [27, 28]. The epidermis and dermis are the two distinct layers that make up the skin. The outermost layer of the skin is the epidermis, which is mainly made up of keratinocytes and non-keratinocytes. Beneath it lies the dermis, the deeper layer of the skin, composed of connective tissue fibers and various cells [29]. The connective tissue in the dermis includes various cells, such as telocytes and fibroblasts. Telocytes are a recently discovered type of interstitial cell, notable for their small cell body and long cytoplasmic extensions. Telocyte was described previously in the dermis of the skin near various important component such as sebaceous and sweat glands, hair follicles, arrector pili muscles, nerves and blood vessels. In addition, telocyte was detected in previously the lamina propria of oral mucosa and underlying skeletal muscle. They create extensive connectivity with many tissue structural elements and are involved in intercellular communication and signaling [21, 30–38]. Additionally, the skin contains appendages like hair, sweat glands, and sebaceous glands [39].

Our research showed that goat skin has various types of encapsulated and non-encapsulated receptors that respond positively to NSE. These types of receptors include Merkel cells, Ruffini’s corpuscles, and free nerve endings. Ruffini’s corpuscles were elongated, fusiform-shaped, encapsulated mechanoreceptors, considered the simplest type. Ruffini’s corpuscles exhibited NSE-positive reactivity [40, 41]. Ruffini corpuscles were detected previously in different animals in the skin and mucous membrane [42] including cattle [43], pig [44], monkey [45], primate [46], and Turtle [47]. Our results were in agreement with Martín-Cruces et al. which described Ruffini corpuscles in human lip [48], and Halata & Munger which described Ruffini corpuscles in primate lip [46].

Merkel cells are non-encapsulated receptors, considered modified epidermal cells located in the stratum basale and associated with hairs. They contain neurosecretory granules in the cytoplasm [41]. NSE was considered a specific marker for Merkel cells [49–51]. Merkel cells have been observed in different regions of skin and mucous membrane of different animal species previously, such as sheep [52], pig [53], dog [54], monkey [55]. Our data agree with Halata et al., which described Merkel cells in the lip of the monkey [53] and Ramírez et al. in lip of dog [54]. On the other hand, Martín-Cruces et al., on the lip of a human, did not find Merkel cell-neurite complexes [48].

The nerve bundles travel through the dermis, creating a network just below the epidermis. At the junction between the dermis and epidermis, they lose their protective Schwann cell covering. Then, they enter the epidermal basement membrane, move between keratinocytes, and finally end as free nerve endings within the epidermis. Free nerve endings of the non-encapsulated simplest receptor type reacted positively to NSE [40, 41].

We found small corpuscles not previously described in animals and named them goat corpuscles. Previously, scientists described Meissner-like corpuscles in the skin of mice [56]. Therefore, new corpuscles in animals are being described in studies every day. Goat corpuscles were small, rounded to oval encapsulated structures located in subepithelial positions and in the dermal reticular layer, particularly near hair follicles and sebaceous glands. Merkel cells can easily differentiated from them because they are associated with the basal layer of the epidermis and are not encapsulated [57, 58]. Also, Ruffini corpuscles can easily distinguished from them through their spindle shaped that lies deeper in the dermis [59]. Free nerve endings are unencapsulated and located in the dermis and epidermis [60]. Krause end-bulbs are located at mucocutaneous junctions and are mainly spherical in shape, and Golgi–Mazzoni corpuscles are located in mucosa and subcutaneous tissue and appear as spherical lamellar structures [60]. So, the goat corpuscles exhibited a unique location and morphology.

Our investigations revealed the presence of NSE not only in the nerve or sensory component, but also in various structural components such as the vessel wall, plasma within the vessels, myoepithelial cells, chondroblasts, chondrocytes and interstitial cells such as fibroblasts and telocytes. Similar observations of NSE expression were detected in the walls of the blood vessels and lymph vessels [61, 62], myoepithelial cells [63, 64], chondrocytes [65], and telocyte [62, 66]. This suggests that these components may function through neuro- and/or neuroendocrine mechanisms.

This study establishes a basis for future investigation into the expression of NSE in both the lip and ear pinna of goat neural and non-neural components. Further research should be performed to explain the importance of NSE expression in different regions of goat skin and during pre- and postnatal development. Moreover, novel findings of the goat corpuscle need to be further clarified using different histological and immunohistochemical, electron microscope and molecular studies to determine its role and its relation. In addition, species comparative studies are needed to elucidate if it is species-specific or not. These directions help to understand the histology and physiology of the skin in goats in relation to other species.

## Data Availability

The datasets utilized and/or examined in the present investigation can be obtained from the corresponding author upon a reasonable request.
